# Locally advanced primary mediastinal atypical carcinoid successfully resected after neoadjuvant treatment: A case report

**DOI:** 10.1111/1759-7714.14091

**Published:** 2021-08-03

**Authors:** Alberto Testori, Vittorio Perfetti, Camilla De Carlo, Paola Bossi, Marco Alloisio, Giuseppe Mangiameli

**Affiliations:** ^1^ Division of Thoracic Surgery IRCCS Humanitas Research Hospital Milan Italy; ^2^ Internal Medicine Unit Oncologia Oltrepò, Ospedale di Varzi, ASST Pavia Pavia Italy; ^3^ Department of Pathology Humanitas Clinical and Research Center – IRCCS Milan Italy; ^4^ Department of Biomedical Sciences Humanitas University Milan Italy

**Keywords:** atypical carcinoid, NETs, neuroendocrine tumor

## Abstract

Neuroendocrine tumors (NETs) are epithelial neoplasms with predominant neuroendocrine differentiation that arise in most organs of the body. Mediastinal NETs are very rare, and account for no more than 5% of all mediastinal tumors. R0 surgery represents the milestone of treatment. Here, we describe a case of a locally advanced primary atypical carcinoid of the mediastinum. This was initially considered inoperable due to infiltration of a great vessel and was successfully resected after neoadjuvant treatment as a result of very extensive surgery. Only through an accurate preoperative diagnosis and good radiological planning is it possible to obtain satisfactory oncological results.

## Graphical abstract

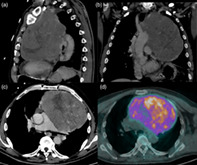

## INTRODUCTION

Neuroendocrine tumors (NETs) are epithelial neoplasms with predominantly neuroendocrine differentiation that arise in most organs of the body.^1^ They are more frequently localized in the gastro‐enterohepatic or respiratory system. NETs of the mediastinum are very rare accounting for no more than 5% of all mediastinal tumors.^2^ Here, we report a case of a primary locally advanced atypical carcinoid (AC) tumor of the mediastinum diagnosed in a patient who underwent neoadjuvant chemotherapy which reduced the size of the mass initially judged to be unresectable and enabled extensive R0 surgery to be performed.

## CASE REPORT

**FIGURE 1:**
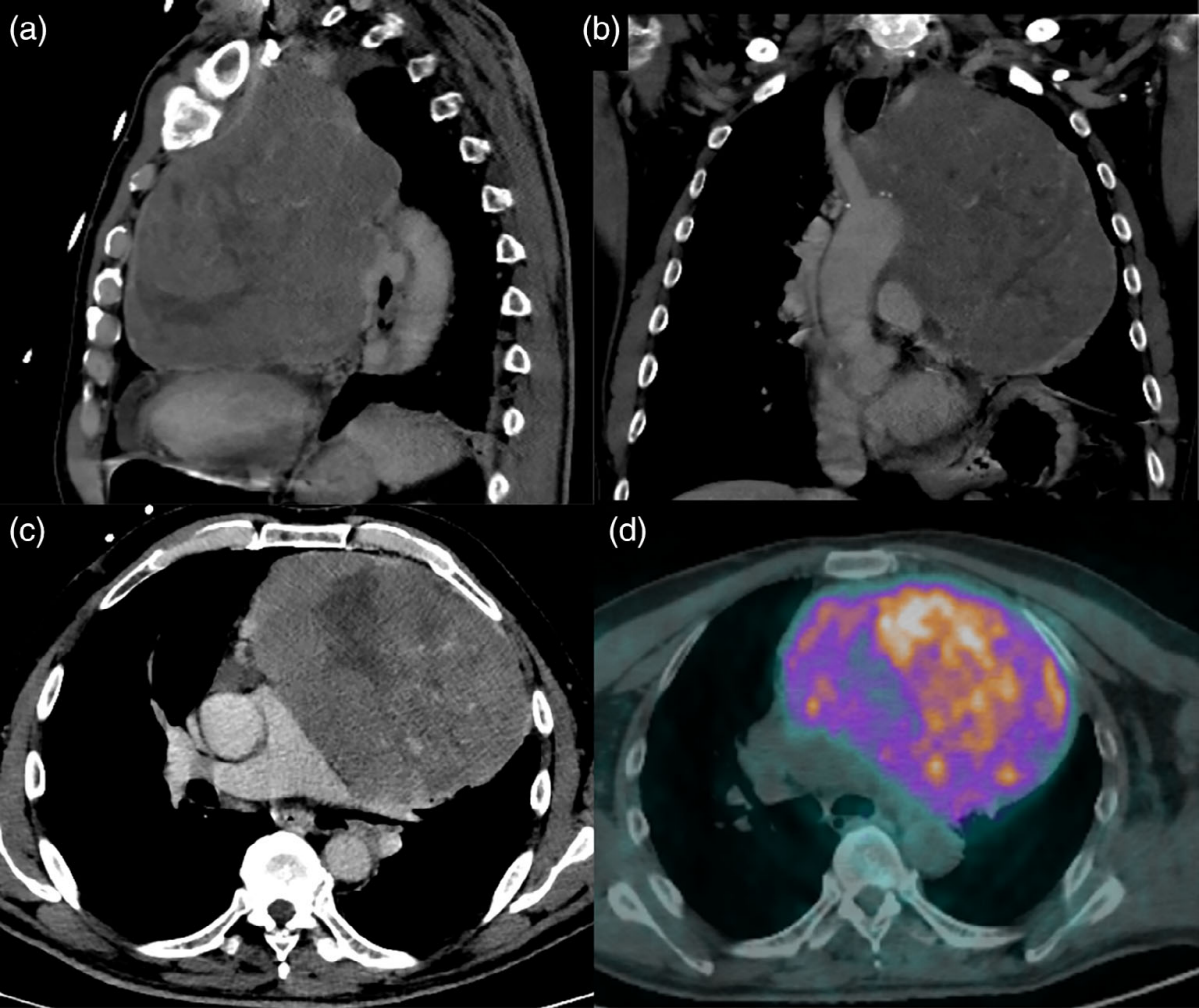
(a,b,c) Sagittal, coronal and axial contrast‐computed tomography (CT) showed the presence of a tumor mass measuring 16 × 12 × 18 cm localized in the anterosuperior mediastinum causing compression of the left cardiac chamber, aortic arch, origin of the supra‐aortic vessels, the common trunk of the pulmonary artery and the left main bronchus. (d) 68Ga‐DOTA‐TOC positron emission tomography (PET) showed high uptake limited to the mediastinal mass

**FIGURE 2:**
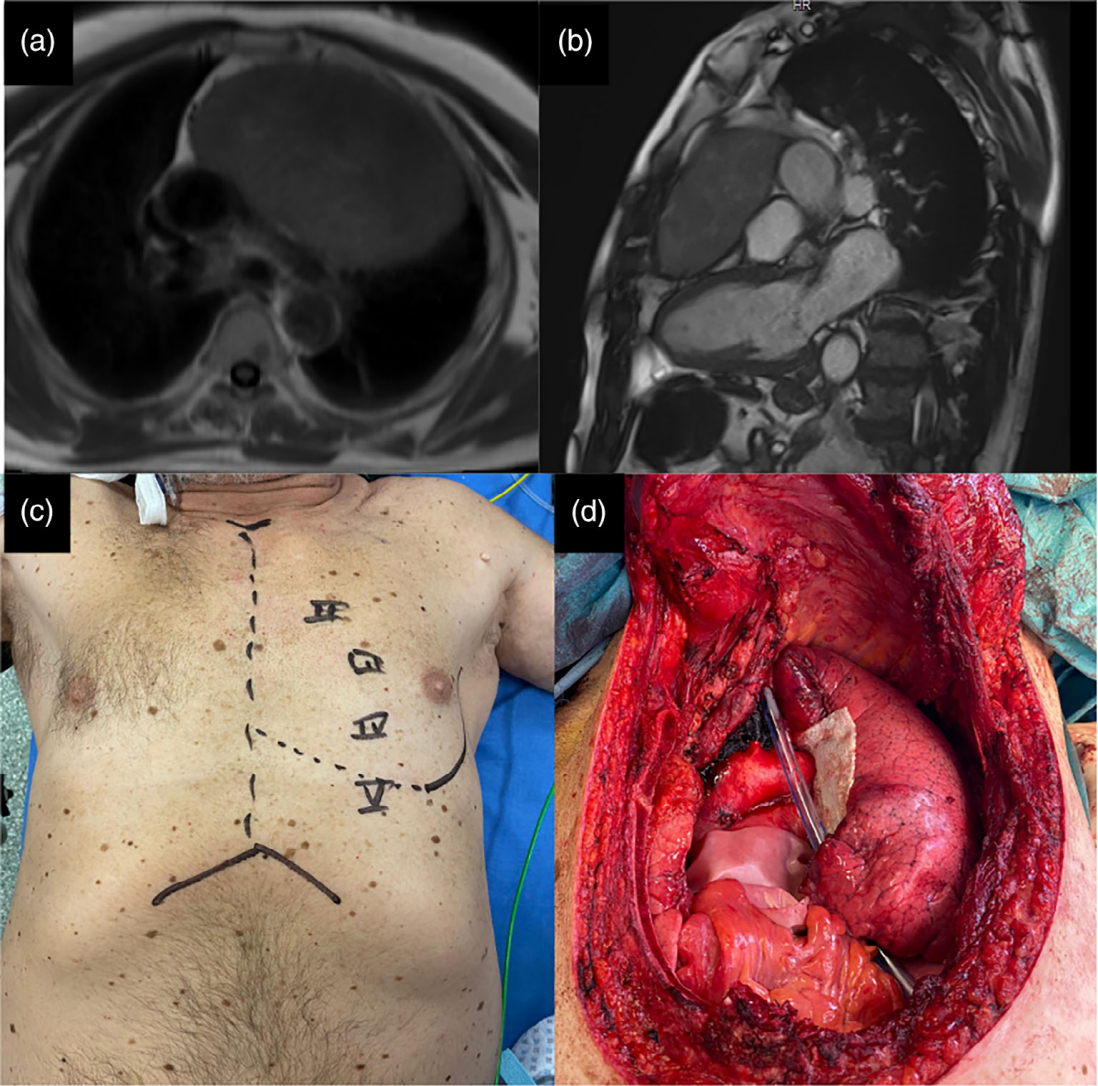
Axial (a) and sagittal (b) slice of a dynamic MRI showed a reduction in size of the mediastinal mass (14 × 8 × 9 cm vs. 16 × 12 × 18 cm) and excluded an infiltration of the common trunk of the pulmonary artery, thoracic aorta and superior cava vein (c) Surgical incision. (d) Surgical postoperative field showing a pulmonary wedge resection of the left upper lobe and a pericardial reconstruction with a bovine pericardium patch after partial pericardiectomy

**FIGURE 3:**
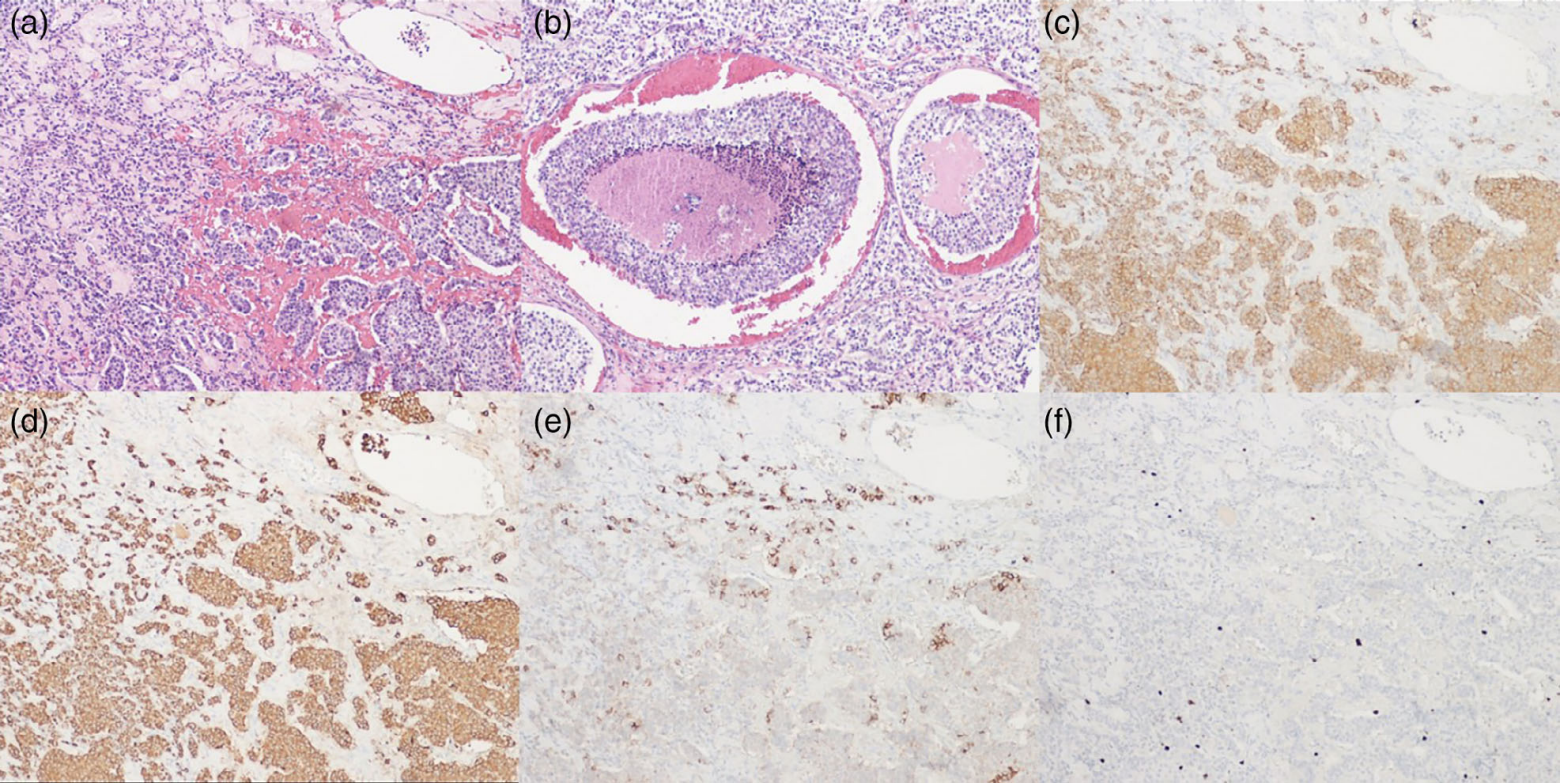
Histopathology of the lesion from the patient in our report (10× magnification). (a) Hematoxylin & eosin (HE) showed a relatively uniform neuroendocrine proliferation of medium‐sized cells with eosinophilic cytoplasm, mild nuclear pleomorphism and small nucleoli with a trabecular pattern of growth and immersed in hyaline stroma with focal necrosis. (b) HE showed central necrosis of the tumor. (c,d) The same field as (a), showing synaptophysin and chromogranin positive cytoplasmic staining, respectively. (e) The same field as (a), showing CD56 positive membranous staining. (f) Ki‐67 brown nuclear staining. The tumor showed three mitoses/2 mmq and the ki‐67 proliferation index was 5%

**FIGURE 4:**
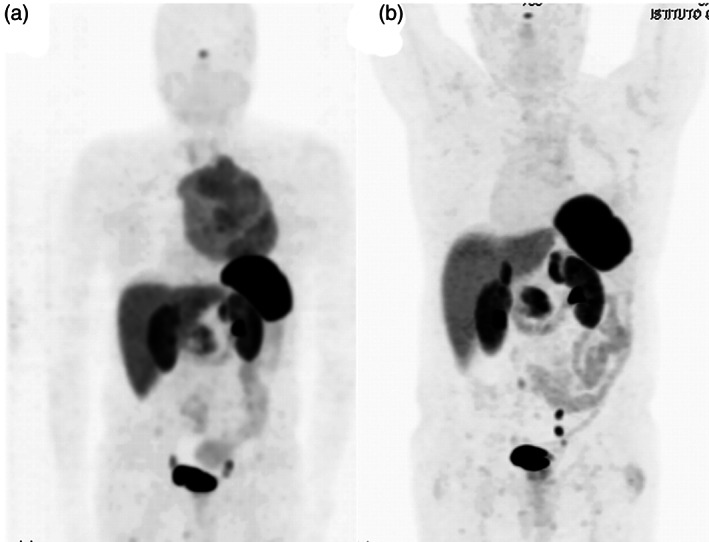
68Ga‐DOTA‐TOC (PET) before neoadjuvant treatment (a) and 6 months after surgery (b)

A 67‐year‐old man, a never smoker, without a significant previous medical history, presented with exertional dyspnea which had worsened in the last two months. There were no associated syndromes on clinical presentation. A chest computed tomography (CT) showed the presence of a mass measuring 16 × 12 × 18 cm in the anterosuperior mediastinum (Figure 1). A DOTA‐D‐Phe^1^‐Tyr3‐octreotide with gallium‐68 (68Ga‐DOTA‐TOC) positron emission tomography (PET) showed high uptake limited to the mediastinal mass (Figure 1). CT‐guided needle biopsy revealed a low grade NET (Ki67 5%) without mitoses and necrosis. After a multidisciplinary meeting, the mass was judged to be unresectable and the patient was scheduled to undergo nine cycles of neoadjuvant chemotherapy with capecitabine and temozolomide (CAPTEM). The chemotherapy regimen was well tolerated by the patient and he was radiologically evaluated every 3 months to establish successive management (surgery in case of reduction in size vs. definitive radiotherapy). A new chest CT‐scan 6 months later shoved a reduction in size of the mass and dynamic heart magnetic resonance imaging (MRI) confirmed the reduction in size, which excluded invasion of the heart or great vessels (Figure 2). A surgical excision was performed through a sternotomy which extended to the left fourth intercostal space. On exploration, the left anonymous vein was partially in contact with the tumor; both apical segments of the left lung and pericardium were largely infiltrated. First, a pulmonary wedge resection was performed after identification of the vagus and recurrent nerve. Second, the superior cava vein was controlled and the left innominate vein was accurately dissected and preserved because no tumor infiltration was detected. Then, the mass was removed en bloc with the pericardium. After partial pericardiectomy, pericardial reconstruction was performed with a bovine pericardium patch (Figure 2). Operative time was 5 h and blood loss was lower than 150 cc. The postoperative course were uneventful. The patient experienced immediate relief of his symptoms and was discharged on postoperative day 13. The histological findings are shown in Figure 3. Immunohistochemical staining confirmed the neuroendocrine nature of the tumor. Clinicopathological findings and the absence of intra‐ and perilesional thymic tissue were suggestive of a primary AC tumor of the mediastinum. Considering the low Ki67 (5%) and R0 resection adjuvant chemotherapy and/or radiation were not performed. Six months after surgery, a 68Ga‐DOTA‐TOC‐PET did not show residual or distant metabolic uptake (Figure 4).

## DISCUSSION

Mediastinal NETs are very rare tumors, and are almost exclusively located in the anterior superior mediastinum, although several cases located in the middle and posterior mediastinum have been previously described in the literature.^2^, ^3^ NETs occurrence is mostly sporadic, associated with syndromes (MEN 1) or family history of malignancies (breast, brain or liver).^4^ Clinically, patients can be asymptomatic and diagnosed during radiological examinations or symptomatic presenting nonspecific symptoms due to the compression or invasion of mediastinal structures, or systemic symptoms (hot, red facial flushing, diarrhea and wheezing) secondary to tumor released hormones or cytokines (lower than 10%).^5^, ^6^

Chest CT scan is essential to define the characteristics of the tumor, its anatomical relationship with surrounding structures and the possibility of obtaining a R0 resection during surgery.^7^, ^8^, ^9^ 68Ga‐DOTATOC PET can provide accurate information on the site and dissemination of the tumor.^4^ If the mass is judged to be unresectable, CT‐guided biopsy is imperative in order to obtain an accurate diagnosis and establish the correct oncological management.

NETs nomenclature traditionally follows that of their pulmonary counterparts. Thus, according to the World Health Organization (WHO) classification, they are separated into typical (low grade) and atypical (intermediate grade) carcinoid tumors, large cell neuroendocrine carcinomas (LCNEC), and small cell carcinomas (SCC).^10^ Typical carcinoid (TC) or AC and large cell neuroendocrine tumors have different prognoses and available treatment options.^11^, ^12^ The 5‐year survival of patients with AC is slightly worse than that of patients with TC, and ranges from 20%–80% in previously reported studies.^13^, ^14^, ^15^

To date, official guidelines for the treatment of thymic NETs are not available. Evidence is based on case reports and case series with a lack of prospective trials. In cases of localized disease, complete surgical resection remains the therapy of choice. R0 resection is significantly associated with a better prognosis compared to tumors submitted to incomplete resection (R1 or R2).^16^ Unfortunately, most NETs are deemed unresectable at the time of diagnosis because approximately half are locally advanced (Masaoka stage III) or metastatic. In locally advanced cases, dynamic heart MRI is often useful to define heart or great vessel infiltration thereby assisting in surgical planning.^8^, ^9^ The role of neoadjuvant chemotherapy or radiation therapy (RT) remains unclear as data is based solely on case reports.^17^ A recent meta‐analysis has confirmed the CAPTEM regimen is effective for treating patients with advanced NET;^18^ in our case, this therapeutic option was effective facilitating R0 surgery.

In conclusion, NETs are very rare epithelial neoplasms. They usually present as a large mass with an infiltrative nature located in the superoanterior mediastinum. Early and accurate diagnosis are very important, and surgery remains the mainstay of treatment. Neoadjuvant chemotherapy should be considered as a possible strategy allowing a RO surgery for locally advanced disease.

## CONFLICT OF INTEREST

The authors declare that there are no conflicts of interest.
